# Supplementary material for: Redox driven B12-ligand switch drives CarH photoresponse
Source: Nat Commun. 2023 Aug 21;14:5082. doi: 10.1038/s41467-023-40817-6 (PMC10442372; doi:10.1038/s41467-023-40817-6)
Supplement: Supplementary file 1 — Supplementary Information [file 41467_2023_40817_MOESM1_ESM.pdf]

# Supplementary Figures and Tables

## Redox driven $B_{12}$-ligand switch drives CarH photoresponse

Harshwardhan Poddar[1], Ronald Rios-Santacruz[2], Derren J. Heyes[1], Muralidharan Shanmugam[3], Adam Brookfield[3], Linus O. Johannissen[1], Colin W. Levy[1], Laura N. Jeffreys[1], Shaowei Zhang[1], Michiyo Sakuma[1], Jacques-Philippe Colletier[2], Sam Hay[1], Giorgio Schiro[2], Martin Weik[2], Nigel S. Scrutton[1*], David Leys[1*]

[1]Manchester Institute of Biotechnology, Department of Chemistry, University of Manchester, United Kingdom

[2]Univ. Grenoble Alpes, CEA, CNRS, Institut de Biologie Structurale, F-38044 Grenoble, France

[3] Photon Science Institute, Department of Chemistry, University of Manchester, United Kingdom

*Corresponding authors: david.leys@manchester.ac.uk; nigel.scrutton@manchester.ac.uk

### Supplementary figures

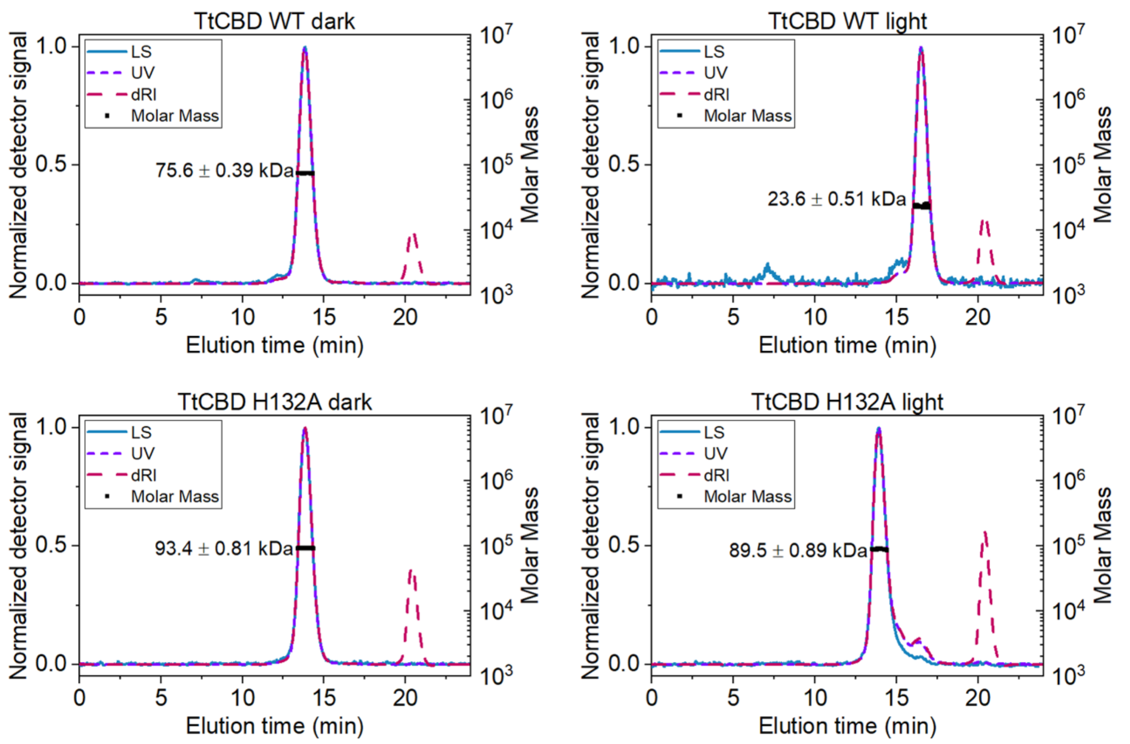


**Supplementary Figure 1**. SEC-MALS analysis of wild type and H132A variant of *Tt*CBD. Chromatogram traces of light scattering (LS), ultraviolet (UV) and differential refractive index (dRI) signals are shown in the figure as solid or dashed line. Molar mass for main peaks is plotted as square scatters. The estimated molecular weight of the main fraction is labelled in the figure.

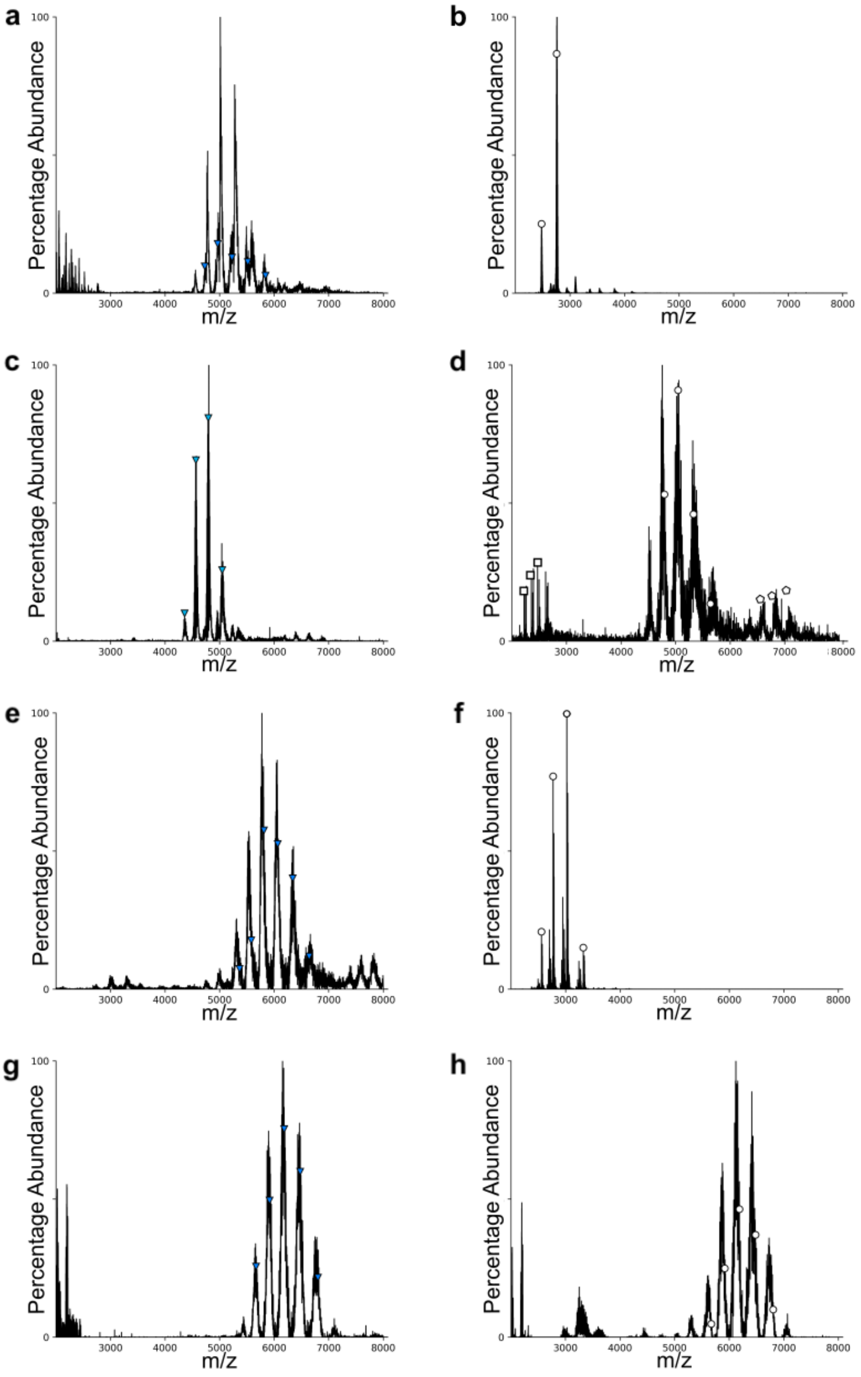


| Protein | Condition | Mass (Da) | Additional masses Observed (Da) | | Calculated masses (Da) |
|---|---|---|---|---|---|
| WT TtCBD | Dark | 99297.8 | | | 99217.1 |
| WT TtCBD | Light | 24792.7 | | | 24554.1 |
| H132A CBD | Dark | 95873.2 | | | 98952.8 |
| H132A CBD | Light | 95869.2 | 45206.4 | 184838.6 | 24505.1 |
| WT full-length TtCarH | Dark | 139483.1 | | | 139406.8 |
| WT full-length TtCarH | Light | 33240.3 | | | 34601.6 |
| H132A full-length | Dark | 136021.0 | | | 139142.6 |
| H132A full-length | Light | 136102.2 | | | 138210.0 |

**Supplementary Figure 2**. Native mass spectrometry of truncated and full-length proteins. a) and b) In response to light WT TtCBD changes mass from 99.3 KDa (panel a) with a predominant charge of +20 to 24.8 KDa (panel b) with a predominant charge of +9 corresponding to a change in conformation from tetramer to monomer. c) and d) The CBD domain of H132A variant was less stable in the ionisation buffer (200 mM ammonium acetate pH 7.0) resulting in multiple states. In the dark state despite low ionisation conditions (0.9 kV 0.2 μA electrospray voltage and current respectively) the predominant peak observed corresponded to a tetramer with a charge of +20 (95.8 KDa in panel c). However, in response to light (panel d) multiple states were observed corresponding to masses of 45.2 KDa (represented by squares) and 183.8 KDa (represented by pentagons), as well as the same species as observed in the dark state but with the charge state +19 (95.8 KDa). e) and f) The full-length WT protein also displayed a change in mass in response to light (139.5 KDa to 33.5 KDa) corresponding to a change from tetramer to monomer and a change from charge state +24 to +11 (panels e and f respectively). g) and h) The full-length H132A variant was more stable than its CBD domain in 200 mM ammonium acetate pH 8 and showed fewer adduct states. However, the mass collected in the dark state still corresponded to a tetramer (136.0 KDa) (panel g). The full-length protein also did not show mass or charge changes in response to exposure to light (panel h). A small peak is observed ~3300 m/z which could not be elucidated and is likely a degradation product. Dark species are shown in blue triangles and light species are shown in white circles. Calculated masses from sequences include the mass of AdoCbl, cobalamin or hydroxocobalamin for respective samples.

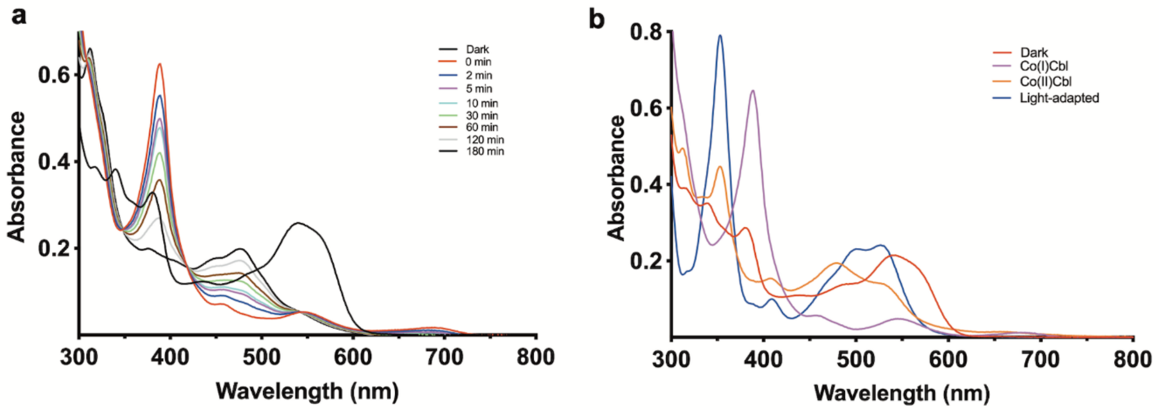


**Supplementary Figure 3**. a) Photoconversion of wild type *Tt*CBD under anaerobic conditions in potassium phosphate buffer pH 7.5. Full conversion of Co(I) to Co(II) is observed in 180 min. The times indicated in the key refer to the length of time samples were incubated in the dark following illumination for 15s. b) Photoconversion of full-length *Tt*CarH under anaerobic conditions showing formation of Co(I) and Co(II) species during the photocycle.

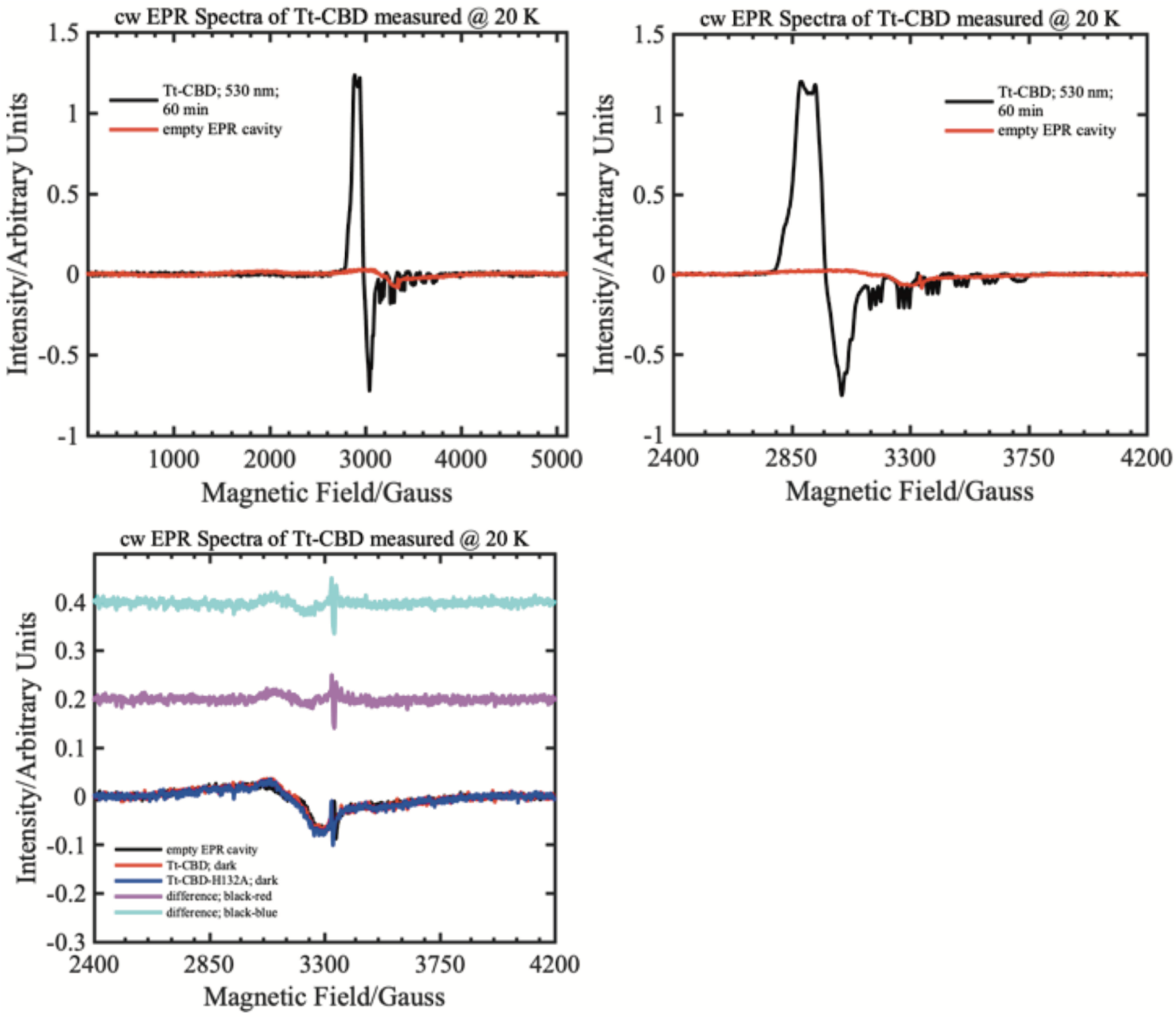


**Supplementary Figure 4**. The top panels show the cw EPR spectra for *Tt*CBD after illumination and incubation in the dark for 60 mins under anaerobic conditions. The bottom panel shows cw EPR spectra for the empty cavity and for EPR silent wild type and H132A dark samples. The sharp EPR signal observed @ 3350 G is likely due to an organic radical (impurity in the EPR cavity) and is present in all the EPR data.

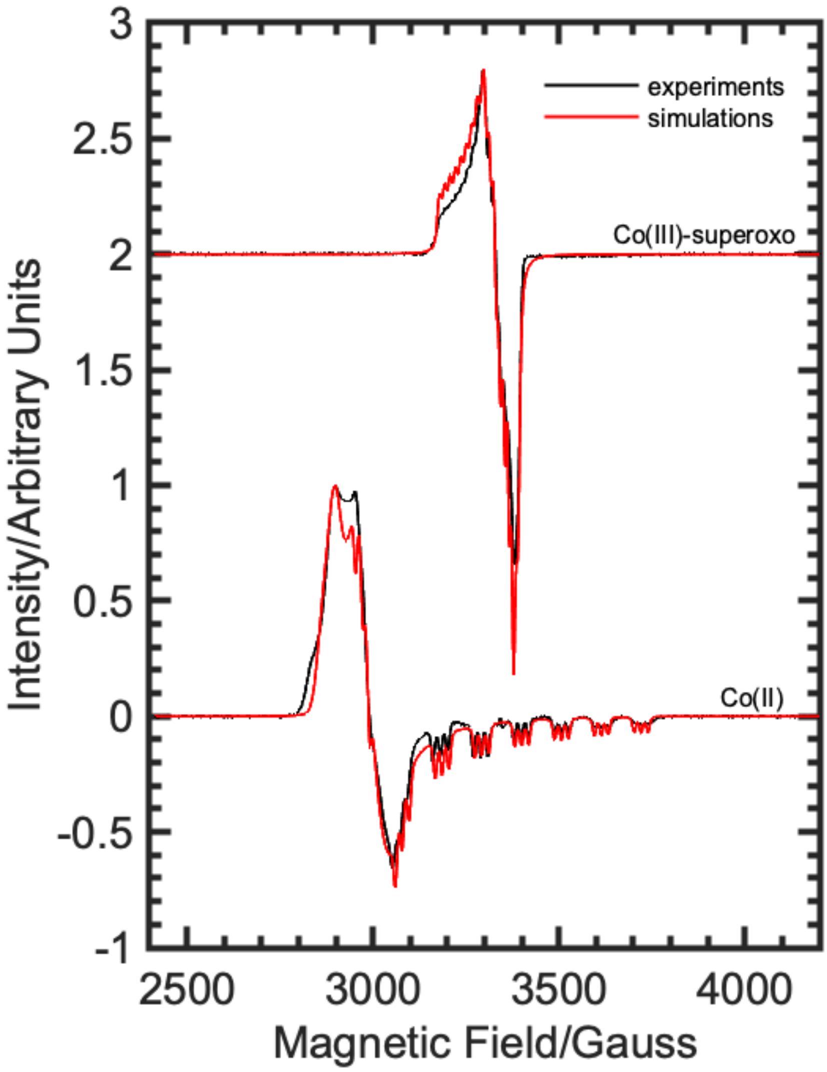


**Supplementary Figure 5**. Experimental cw-EPR spectra (black traces) of the Co(II) (bottom trace) and Co(III)-super-oxo (top trace) species of *Tt*CBD enzyme measured at 20 K as a frozen solution. The simulations (red traces) are overlaid for a comparison. The spin-Hamiltonian parameters used to model the EPR spectra are given below; Co(II)-species – **g** = [2.003 2.216 2.259], **A**($^{59}$Co) = [300 20 15] MHz, **A**($^{14}$N) = [52 36 52] MHz, line widths = [0.37 0.2] mT and HStrain= [20 110 100] MHz; Co(III)-super-oxo species – **g** = [1.998 2.002 2.075], **A**($^{59}$Co) = [17 35 42] MHz, line widths = [0.54 0.48] mT and HStrain= [0 0 15] MHz. The extracted g- and hyperfine tensors agree with the reported values (references; *Coord. Chem. Rev.,* **1981**, 39, 295, *J. Am. Chem. Soc.,* **2012**, 134, 796, *J. Am. Chem. Soc.,* **2016**, 138, 14186, *J. Am. Chem. Soc.,* **2012**, 141, 10984, *Appl. Magn. Reson.,* **2001**, 20, 35).

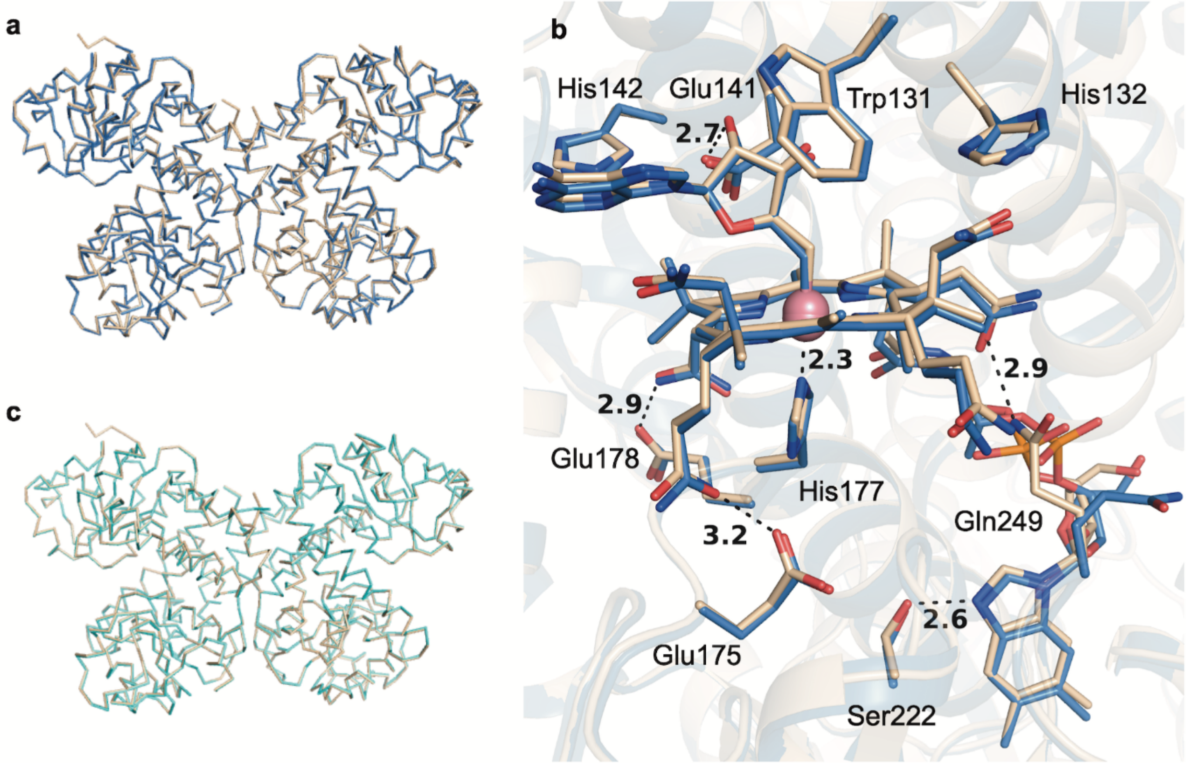


**Supplementary Figure 6**. a) Superposition of the form 1 *Tt*CBD $D^{O2}$ structure (beige) with the CBD of full length *Tt*CarH (blue, pdb id 5C8D). b) Comparison of AdoCbl binding in the aerobic form 1 TtCBD $D^{O2}$ structure and in 5C8D showing conserved AdoCbl binding in the chromophore binding pocket. c) Superposition of form 1 *Tt*CBD $D^{O2}$ (beige) and $D^{anaer}$ (cyan) structures showing hardly any differences in the overall structure in absence of $O_2$.

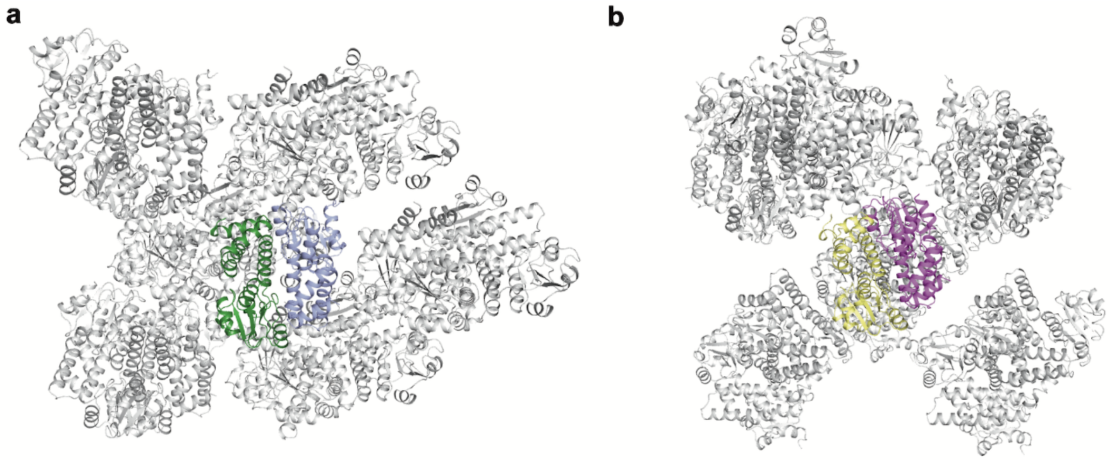


**Supplementary Figure 7.** Cartoon representation of the symmetry elements in dimers a) A-B and b) C-D of the form 1 $D^{O2}$ structure. The crystal contacts within 10 Å of the structure are shown in grey.

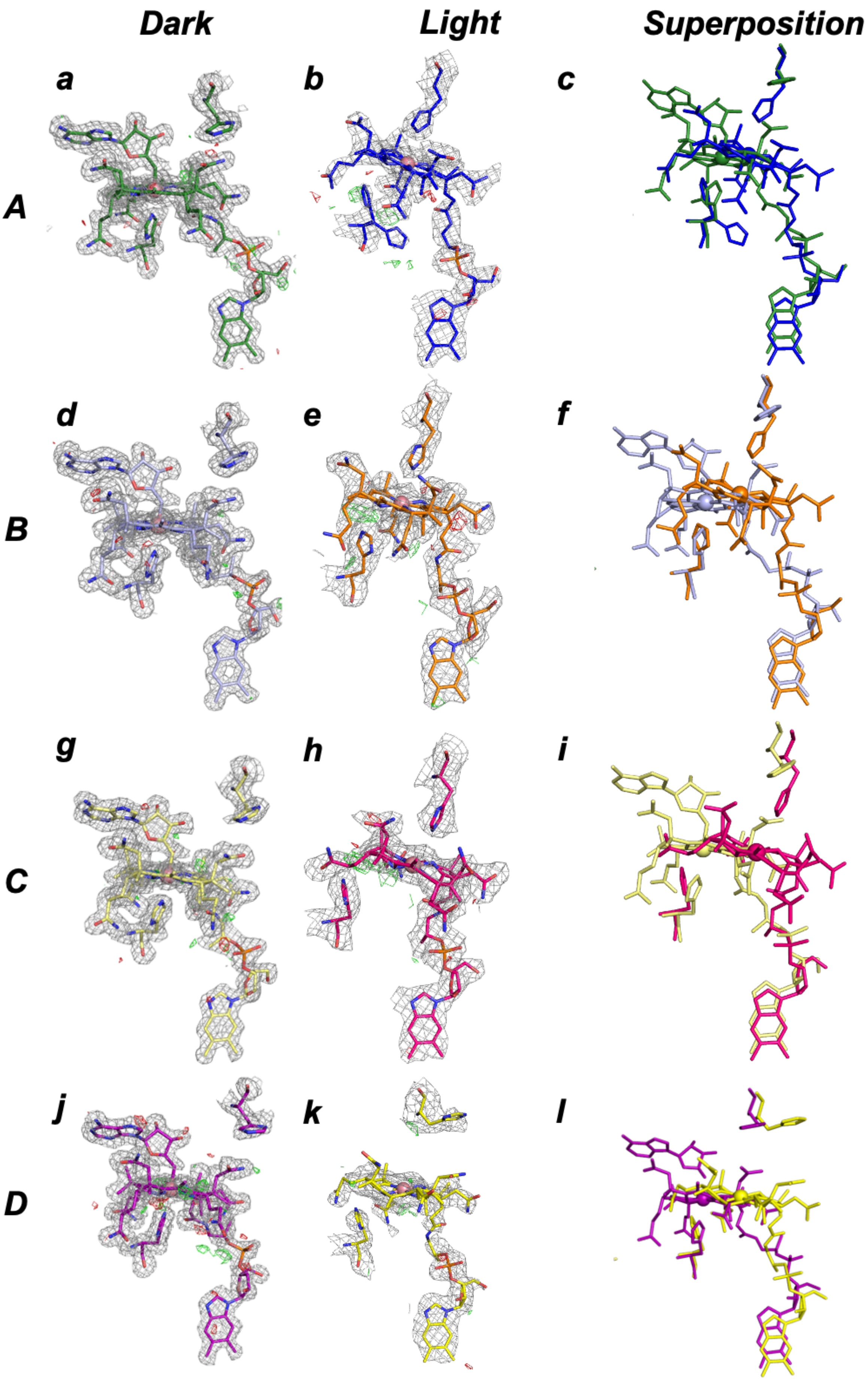
Dark
Light
Superposition
a
b
c
A
d
e
f
B
g
h
i
C
j
k
l
D

**Supplementary Figure 8:** Photoconversion of wild type *Tt*CBD form 2 crystals *in crystallo* under aerobic conditions. The $2F_o$-$F_c$ (grey, 1 σ) and $F_o$-$F_c$ (red (-3 σ), green (+3 σ) electron density maps are shown for the $D^{O2}$ structure (*a,d,g,j*) and the $I^{O2}$ structure obtained after 5 s illumination of *Tt*CBD form 2 crystals at RT under aerobic conditions (*b,e,h,k*). Models of the AdoCbl chromophore, His132 and His177 in monomers A, B, C and D are shown as sticks, the cobalt atom as a sphere. (*c,f,i,l*) Superpositions of dark (green, grey, yellow, magenta) and illuminated (blue, orange, red, yellow) models after respective alignments of monomer-A, -B, -C and -D models with the *super* algorithm from *PYMOL.*

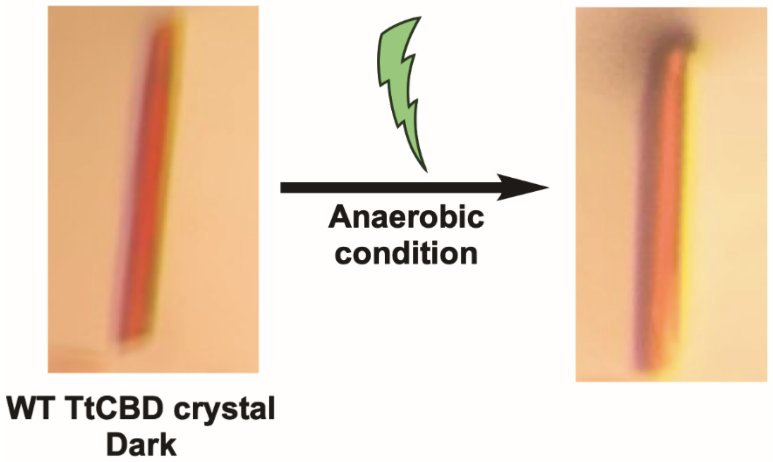


**Supplementary Figure 9.** Photoconversion as observed for wild type TtCBD *in crystallo*. The bright pink "dark" form 1 crystal turns pale orange in response to light illumination under anaerobic conditions.

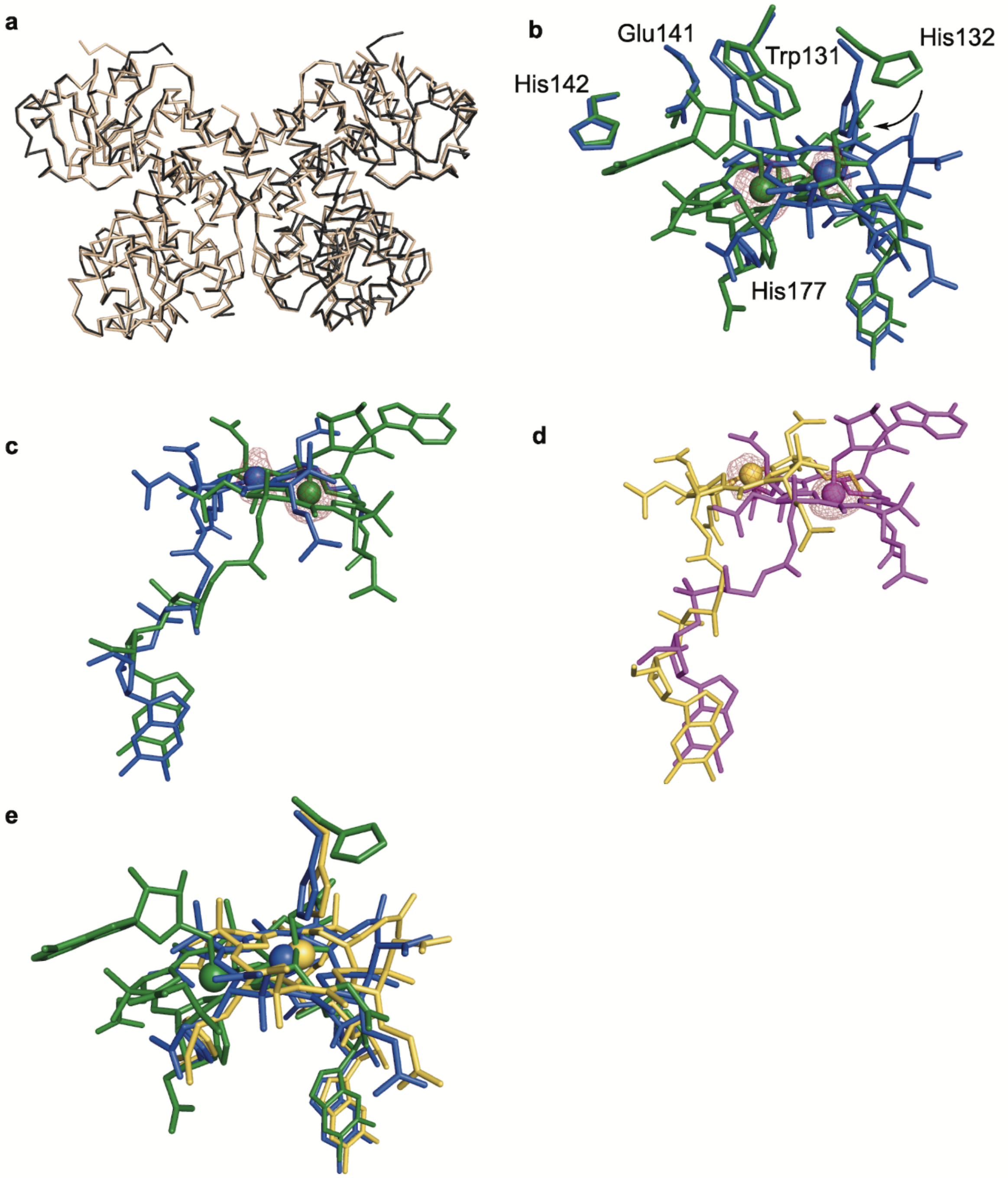


**Supplementary Figure 10.** a) Superposition of form 1 *Tt*CBD $D^{anaer}$ (beige) and $I^{anaer}$ (black) structures. b) and c) different views of cobalamin binding in chain A of $D^{anaer}$ (green) and $I^{anaer}$ (blue) structures. The red mesh shows the anomalous density for Co atom contoured at 3.0 σ. d) comparison of cobalamin binding in chain D of $D^{anaer}$ (purple) and $I^{anaer}$ (yellow) structures. e) comparison of cobalamin binding in chains A (blue) and D (yellow) with respect to $D^{anaer}$ (green) structure.

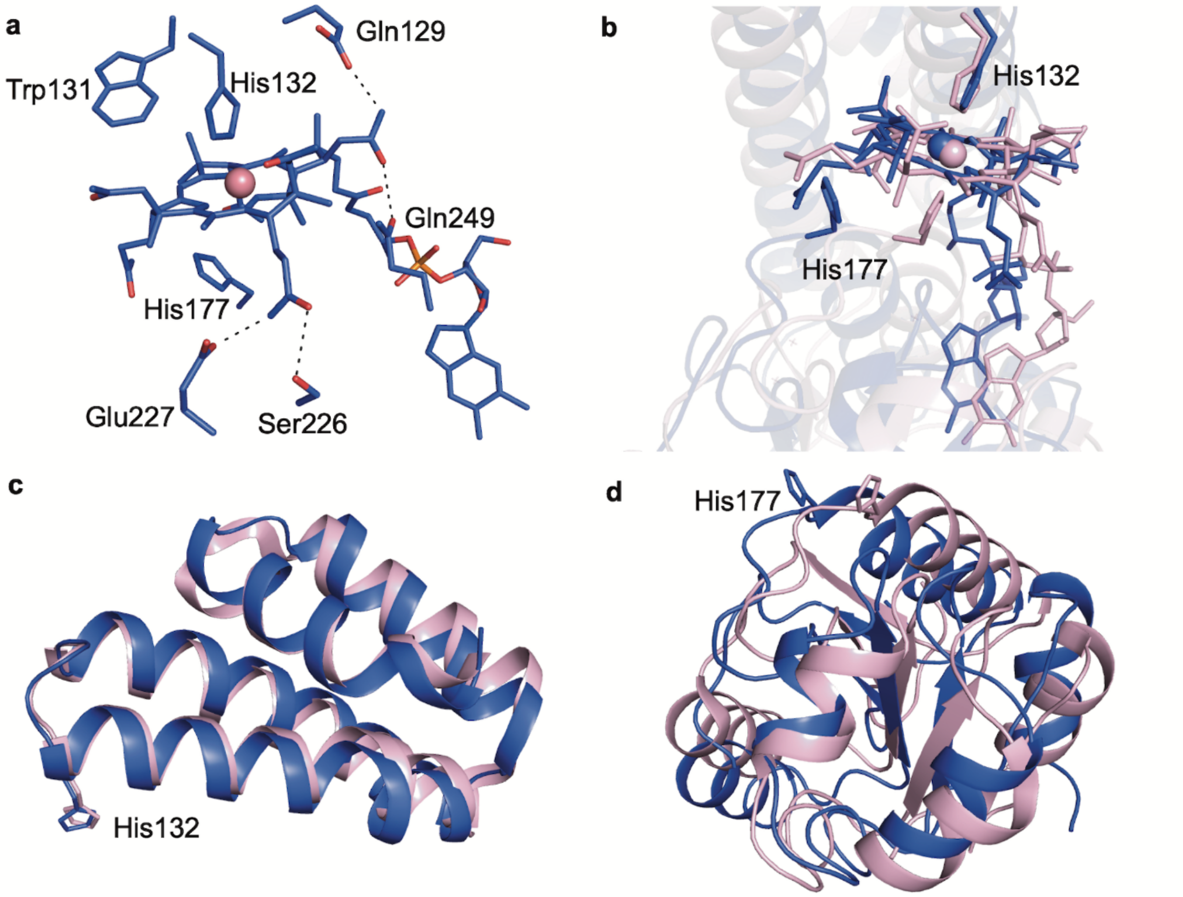


**Supplementary Figure 11.** Comparison of illuminated wild type form 1 $I^{anaer}$ structure (blue) with bis-His ligated light adapted state (pink, pdb id 5C8F). a) Hydrogen bonding interactions formed by amides of the corrin ring in the displaced cobalamin. b) Overlay of wild type light illuminated $I^{anaer}$ structure and light adapted monomer showing the location of bound cobalamin after Co-C bond cleavage. Cartoon representation of overlay of the c) four helix bundle domain and d) Rossmann fold.

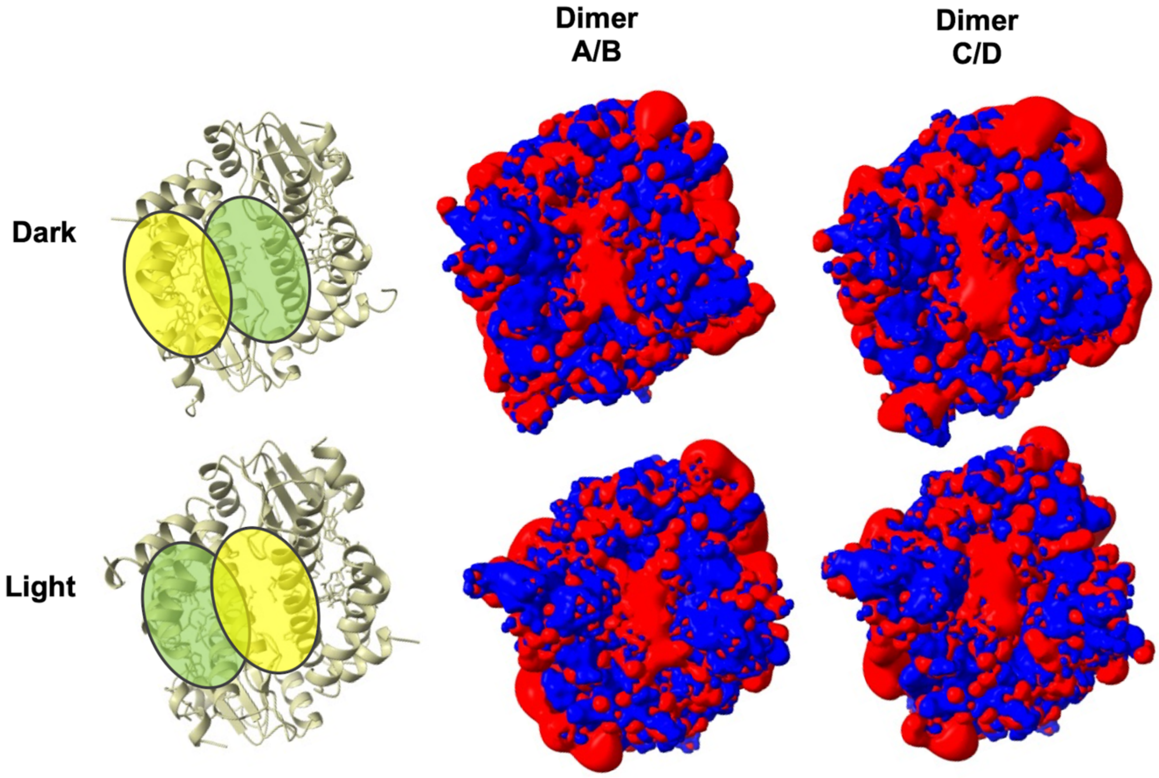


**Supplementary Figure 12.** Electrostatic potential maps for the A/B and C/D dimers of the form 1 $D^{anaer}$ and $I^{anaer}$ structures. The ribbon structures on the left illustrate approximately how the dimers fit together: residues within the yellow circle of one dimer sit on top of the residues within the green circle of the other dimer. There is a high level of electrostatic complementarity at the dimer interface.

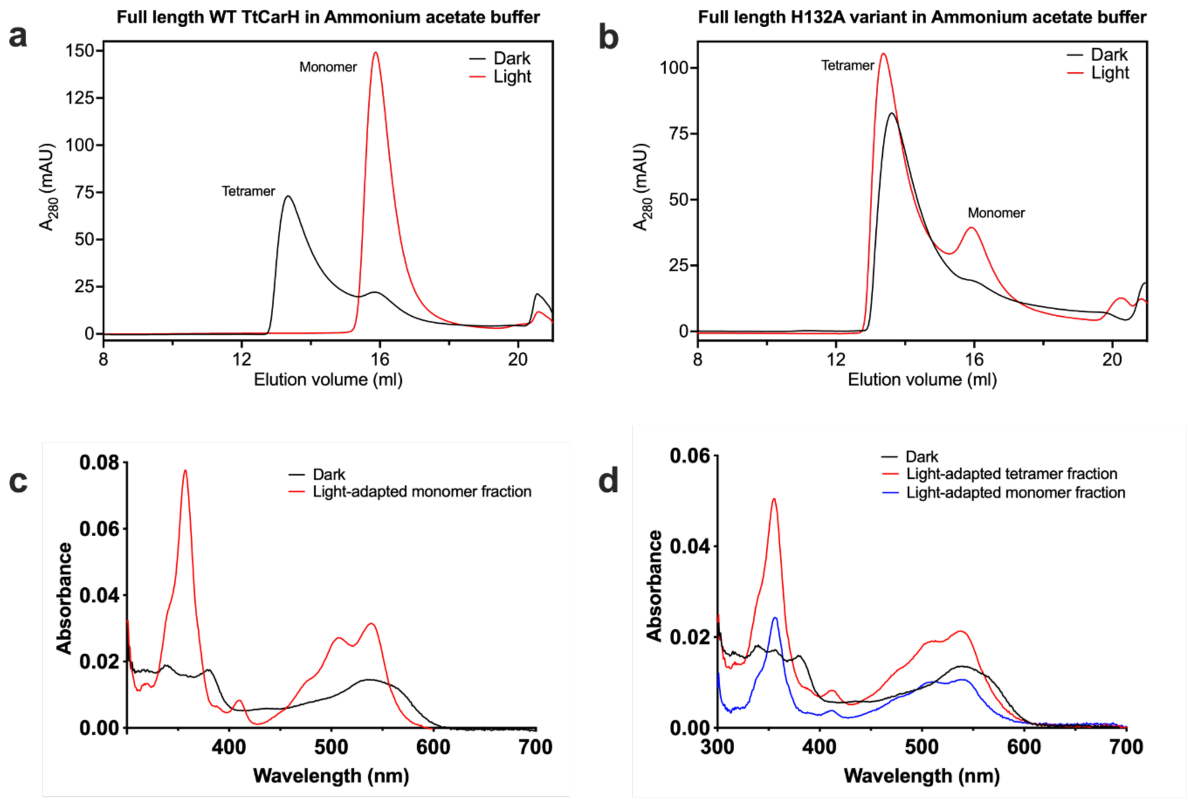


**Supplementary Figure 13**. Elution profile of dark and light-adapted a)WT full-length TtCarH and b) full-length H132A variant from a Superdex200 10/300 GL column using 200 mM ammonium acetate pH 8.0 as an eluent. UV-Vis absorbance spectra of fractions eluting as tetramer or monomer for c) WT full-length TtCarH and d) full-length H132A variant.

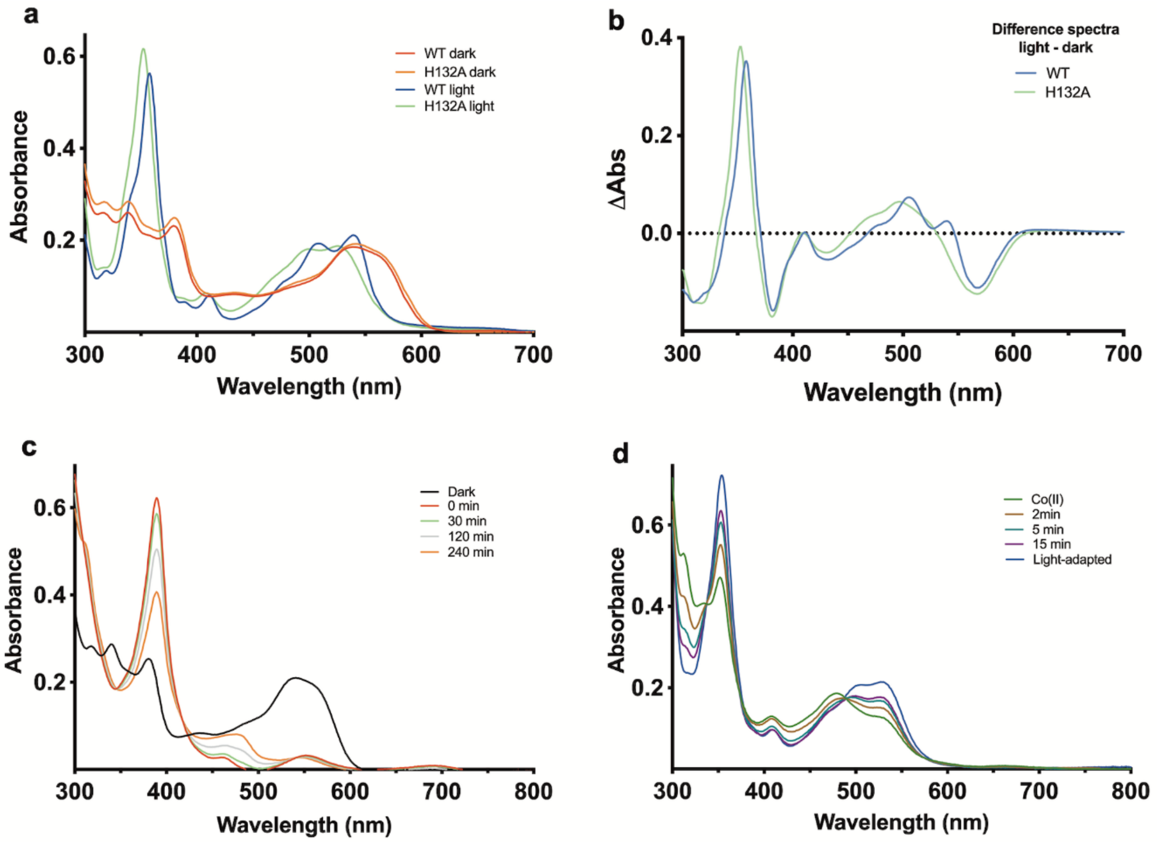


**Supplementary Figure 14.** UV-Vis spectroscopy of *Tt*CBD-H132A . a) Comparison of dark and light states of H132A variant with wild type *Tt*CBD. b) Difference spectra for wild type and H132A variant. C) photoconversion of H132A variant under anaerobic condition in potassium phosphate buffer pH 7.5. Significant amounts of Co(I) species is still observed even after 240 min. The 0 min spectra refers to the first measurement immediately following illumination. d) Conversion of Co(II) H132A variant to light adapted state after exposure to $O_2$.

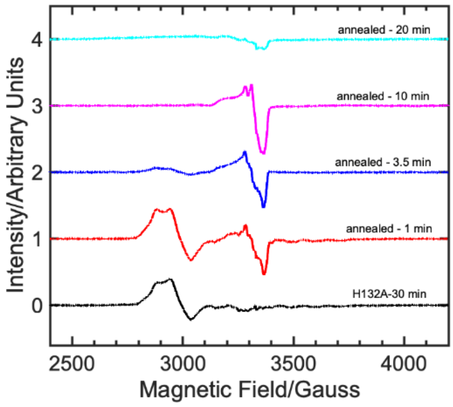


**Supplementary Figure 15**. Formation of additional EPR signals associated with Co(III)-super-oxo species is observed when the *Tt*CBD-H132A samples were exposed to atmospheric oxygen.

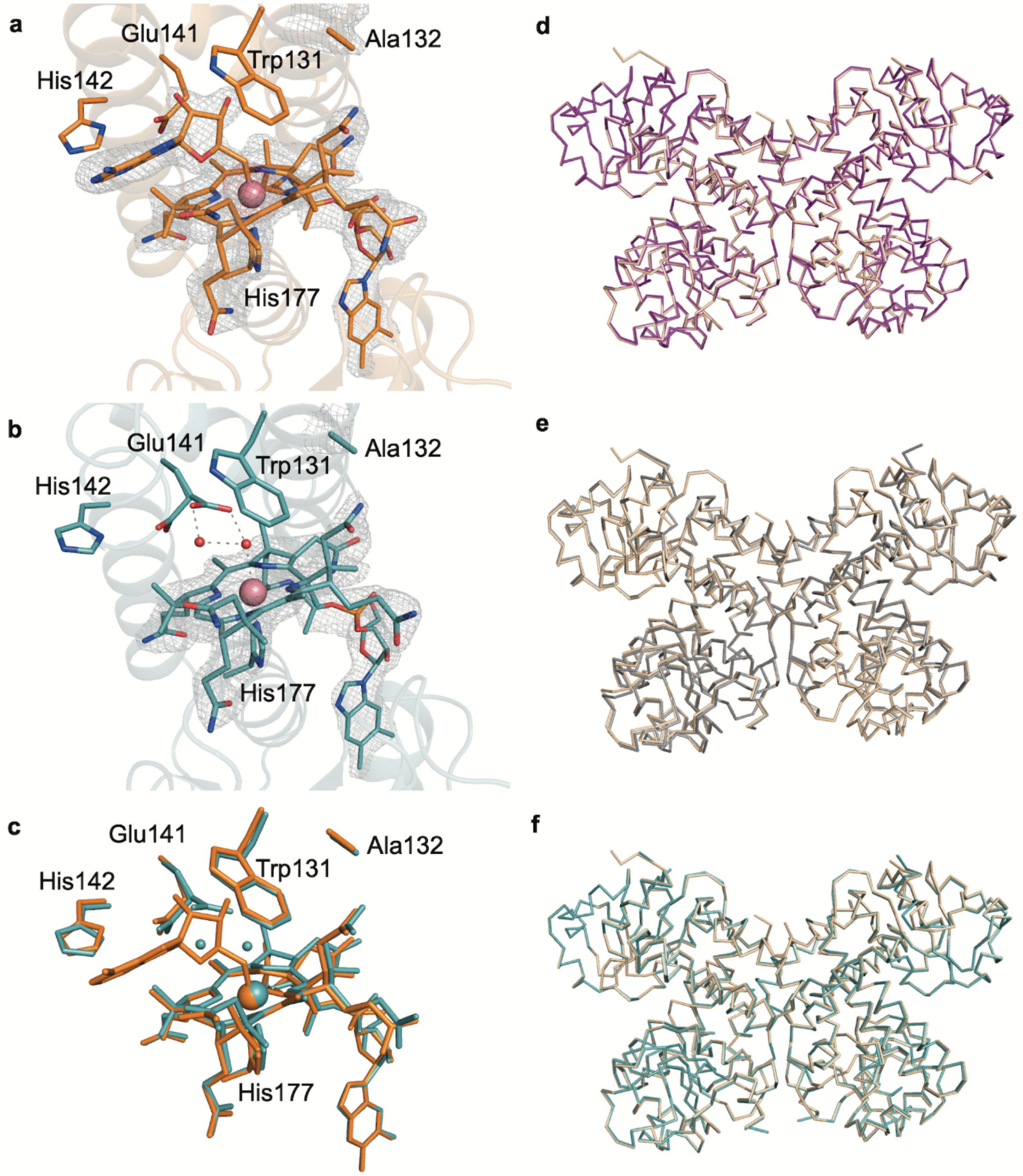


**Supplementary Figure 16**. Figure showing the electron density for bound a) AdoCbl in $D^{O2\text{-}H132A}$ and b) Cbl in $I^{O2\text{-}H132A}$ structures. The grey mesh depicts the composite omit 2Fo-Fc density contoured at 1.5 σ. The hydrogen bonding interactions are shown as dashed lines. C) Overlay of chromophore binding pocket in $D^{O2\text{-}H132A}$ (orange) and $I^{O2\text{-}H132A}$ (teal) structures. Superposition of TtCBD $D^{O2}$ (beige) with d) $D^{O2\text{-}H132A}$ (purple), e) $I^{anaer\text{-}H132A}$ (grey) and f) $I^{O2\text{-}H132A}$ structures (teal).

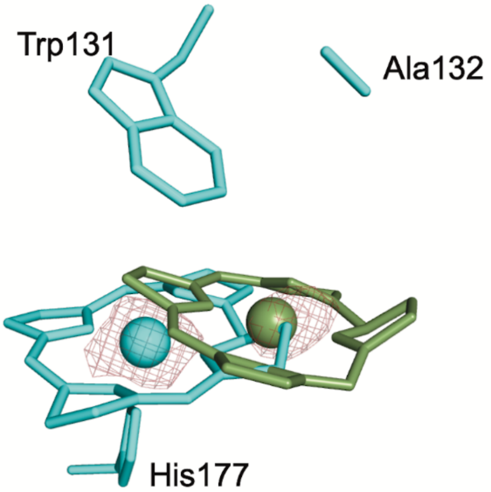


**Supplementary Figure 17**. Two binding modes for cobalamin in chain D of $I^{anaer\text{-}H132A}$ structure. The red mesh shows the anomalous density for Co atom contoured at 3.0 σ.

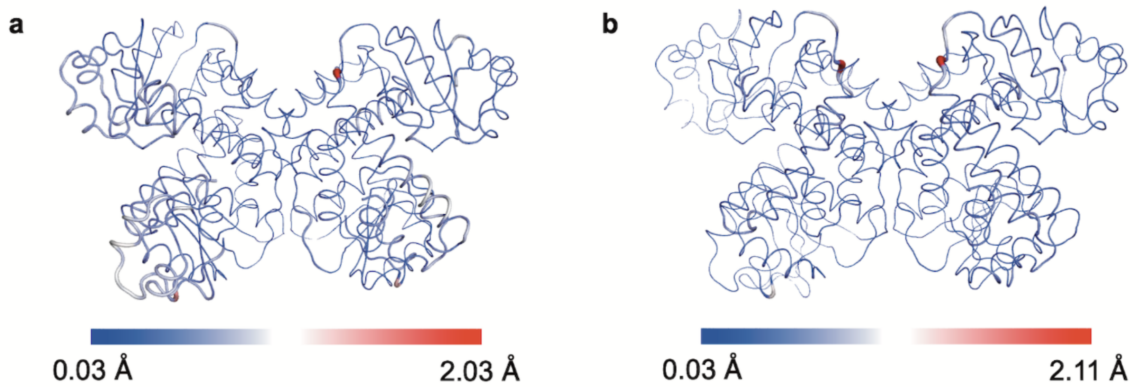


**Supplementary Figure 18**. Figure showing the displacement for Cα atoms belonging to residue 83 to 265 in the a) $I^{anaer\text{-}H132A}$ and b) $I^{O2\text{-}H132A}$ structure with respect to the $D^{O2\text{-}H132A}$ structure.

**Supplementary Table 1**. Data collection and refinement statistics for form 1 crystals

| | **Wild type dark Aerobic ($D^{O2}$)** | **Wild type dark Anaerobic ($D^{anaer}$)** | **Wild type light Anaerobic ($I^{anaer}$)** | **Wild type light Aerobic ($I^{O2}$)** | **H132A dark Aerobic ($D^{O2-H132A}$)** | **H132A light Aerobic ($I^{O2-H132A}$)** | **H132A light Anaerobic ($I^{anaer-H132A}$)** |
|---|---|---|---|---|---|---|---|
| **PDB ID** | **8C31** | **8C32** | **8C33** | **8C34** | **8C35** | **8C36** | **8C37** |
| **Data collection** | | | | | | | |
| Space group | $P\ 2_12_12_1$ | $P\ 2_12_12_1$ | $P\ 2_12_12_1$ | $P\ 2_12_12_1$ | $P\ 2_12_12_1$ | $P\ 2_12_12_1$ | $P\ 2_12_12_1$ |
| Cell dimensions | | | | | | | |
| *a*, *b*, *c* (Å) | 64.1, 69.7, 204.5 | 63.7, 69.4, 204.1 | 60.9, 72.6, 200.9 | 60.9, 72.7, 201.6 | 64.0, 70.0, 204.5 | 63.4, 70.7, 203.8 | 61.8, 69.7, 201.8 |
| α β γ (°) | 90.0, 90.0, 90.0 | 90.0, 90.0, 90.0 | 90.0, 90.0, 90.0 | 90.0, 90.0, 90.0 | 90.0, 90.0, 90.0 | 90.0, 90.0, 90.0 | 90.0, 90.0, 90.0 |
| Resolution (Å) | 61.18 – 1.80 | 57.40 – 2.20 | 60.92 – 2.25 | 52.10 – 1.82 | 54.21 – 2.10 | 67.90 – 2.00 | 61.82 – 2.15 |
| $R_{merge}$ | 0.086 (1.461) | 0.107 (2.095) | 0.206 (1.382) | 0.101 (2.133) | 0.142 (1.382) | 0.141 (2.236) | 0.089 (1.261) |
| $I / \sigma I$ | 16.7 (1.0) | 14.0 (1.4) | 9.4 (1.2) | 19.0 (1.1) | 11.2 (1.7) | 10.3 (1.0) | 18.2 (1.0) |
| Completeness (%) | 98.4 (96.7) | 100.0 (100.0) | 100.0 (98.4) | 100.0 (97.4) | 100.0 (100.0) | 100.0 (100.0) | 100.0 (100.0) |
| Redundancy | 13.6 (14.1) | 12.3 (13.6) | 13.4 (13.3) | 25.7 (15.9) | 13.5 (12.7) | 13.5 (13.5) | 13.4 (13.7) |
| CC1/2 | 1.0 (0.8) | 1.0 (0.7) | 1.0 (0.7) | 1.0 (0.5) | 1.0 (0.8) | 1.0 (0.6) | 1.0 (0.8) |
| **Refinement** | | | | | | | |
| Resolution (Å) | 61.18 – 1.80 | 57.38 - 2.20 | 58.93 – 2.25 | 52.10 – 1.82 | 51.19 2.10 | 66.85 – 2.00 | 59.18 – 2.15 |
| No. reflections | 84402 | 46878 | 43218 | 81010 | 54531 | 62821 | 48408 |
| $R_{work}$ / $R_{free}$ | 17.0/20.9 | 17.9/22.3 | 19.8/24.8 | 16.6/19.8 | 18.6/22.3 | 18.1/21.8 | 19.3/23.0 |
| No. atoms | | | | | | | |
| Protein | 5978 | 5959 | 6046 | 6116 | 5808 | 5941 | 5935 |
| Ligand/ion | 479 | 437 | 365 | 404 | 436 | 364 | 455 |
| Water | 490 | 388 | 331 | 659 | 435 | 475 | 341 |
| *B*-factors | | | | | | | |
| Protein | 46.46 | 64.96 | 41.17 | 37.68 | 46.07 | 49.21 | 54.41 |
| Ligand/ion | 43.03 | 61.01 | 50.25 | 47.87 | 42.22 | 47.64 | 65.78 |
| Water | 50.54 | 61.05 | 41.76 | 50.14 | 51.94 | 54.71 | 58.09 |
| R.m.s. deviations | | | | | | | |
| Bond lengths (Å) | 0.011 | 0.010 | 0.010 | 0.011 | 0.010 | 0.015 | 0.021 |
| Bond angles (°) | 2.11 | 2.00 | 2.07 | 2.09 | 2.04 | 2.10 | 2.32 |

Values in brackets are for the highest resolution shell

**Supplementary Table 2.** Data collection and refinement statistics for form 2 crystals

| | **Wild type dark aerobic ($D^{O2}$)** | **Wild type light aerobic ($I^{anaer}$)** |
|---|---|---|
| **PDB ID** | **8C73** | **8C76** |
| **Data collection** | | |
| Space group | P $2_12_12_1$ | P $2_12_12_1$ |
| Cell dimensions | | |
| *a*, *b*, *c* (Å) | 64.5, 66.5, 205.2 | 61.8, 73.1, 204.8 |
| α β ɣ (°) | 90.0, 90.0, 90.0 | 90.0, 90.0, 90.0 |
| Resolution (Å) | 29.91 – 1.70 | 29.89 – 2.50 |
| $R_{merge}$ | 0.032 (0.493) | 0.033 (0.443) |
| $I / \sigma I$ | 13.8 (1.3) | 11.8 (1.3) |
| Completeness (%) | 99.6 (99.5) | 99.8 (99.9) |
| Redundancy | 2.0 (2.0) | 2.0 (2.0) |
| CC1/2 | 0.999 (0.69) | 0.998 (0.677) |
| | | |
| **Refinement** | | |
| Resolution (Å) | 29.91 – 1.70 | 29.89 - 2.50 |
| No. reflections | 194278 | 65587 |
| $R_{work}$ / $R_{free}$ | 16.7/20.7 | 19.6/22.8 |
| No. atoms | | |
| Protein | 6092 | 6092 |
| Ligand/ion | 436 | 364 |
| Water | 914 | 141 |
| *B*-factors | | |
| Protein | 33.15 | 74.09 |
| Ligand/ion | 29.30 | 91.82 |
| Water | 47.87 | 69.11 |
| R.m.s. deviations | | |
| Bond lengths (Å) | 0.012 | 0.011 |
| Bond angles (°) | 2.11 | 2.07 |

Values in brackets are for the highest resolution shell

**Supplementary Table 3**. Average displacement of Cα atoms in different chains with respect to the wild type form 2 dark structure ($D^{O2}$)

| Structure | | Average displacement per chain for Cα atoms(Å) | | | |
|---|---|---|---|---|---|
| | | A | B | C | D |
| WT_Anaerobic_light ($I^{anaer}$) | Four helix bundle | 0.79 | 0.56 | 0.61 | 1.03 |
| | Rossmann fold | 0.46 | 0.61 | 0.64 | 1.05 |
| H132A_Anaerobic_light ($I^{anaer-H132A}$) | Four helix bundle | 0.40 | 0.35 | 0.36 | 0.42 |
| | Rossman fold | 0.53 | 0.46 | 0.40 | 0.43 |
| H132A_Aerobic_light ($I^{O2-H132A}$) | Four helix bundle | 0.30 | 0.41 | 0.31 | 0.41 |
| | Rosmann fold | 0.21 | 0.24 | 0.27 | 0.38 |

**Supplementary Table 4**. Binding free energies (Δ$G$) and electrostatic forces ($E_{elec}$) between monomers in each dimer and between dimers in the tetramer of $D^{O2}$ and $I^{anaer}$ structures obtained from form 2 crystals

| | Δ$G$ (kcal $mol^{-1}$) | | | $F_{elec}$ (kT $Å^{-1}$) | | |
|---|---|---|---|---|---|---|
| | Dark | Light | % Change | Dark | Light | % Change |
| A-B | -138 | -102 | 31.6 | 80.4 | 55.0 | -26.0 |
| C-D | -115 | -124 | -7.70 | 66.9 | 50.4 | -24.6 |
| AB-CD | -168 | -106 | 36.6 | 141 | 47.8 | -66.2 |
