# Supplementary material for: Redox driven B12-ligand switch drives CarH photoresponse
Source: Nat Commun. 2023 Aug 21;14:5082. doi: 10.1038/s41467-023-40817-6 (PMC10442372; doi:10.1038/s41467-023-40817-6)
Supplement: Supplementary file 3 — Description of additional supplementary files [file 41467_2023_40817_MOESM3_ESM.pdf]

Description of Additional Supplementary Files

Supplementary Data 1: SEC_MALS. Source data for all SEC_MALS traces for different samples.

Supplementary Data 2: Electrostatic_forces_Delphi. Source data for electrostatic forces and binding energies for light and dark state *Tt*CBD dimers and tetramer structures, calculated using the DelPhi web server Delphi.

Supplementary Movie 1: Conformational changes in cobalamin binding and protein backbone of CBD region of *Tt*CarH following light activation. The PDB codes used to generate the movie are as follows: 8C31 (Dark state, tetramer), 8C37 (His177-Co bond breakage and Co(I) oxidation state, intermediate tetramer), 8C33 (cobalamin displacement, His132-Co ligation and Co(II) oxidation state, intermediate tetramer) and 5C8F (bis-His ligated light adapted monomer). The movie was created using a combination of Pymol Morph and Blender.
