# Supplementary material for: Redox driven B12-ligand switch drives CarH photoresponse
Source: Nat Commun. 2023 Aug 21;14:5082. doi: 10.1038/s41467-023-40817-6 (PMC10442372; doi:10.1038/s41467-023-40817-6)
Supplement: Supplementary file 7 — Reporting Summary [file 41467_2023_40817_MOESM7_ESM.pdf]

nature portfolio

Corresponding author(s): David Leys and Nigel Scrutton

Last updated by author(s): 2023/08/03

Reporting Summary

Nature Portfolio wishes to improve the reproducibility of the work that we publish. This form provides structure for consistency and transparency in reporting. For further information on Nature Portfolio policies, see our Editorial Policies and the Editorial Policy Checklist.

Statistics

For all statistical analyses, confirm that the following items are present in the figure legend, table legend, main text, or Methods section.

n/a	Confirmed	
☒	☐	The exact sample size (n) for each experimental group/condition, given as a discrete number and unit of measurement
☒	☐	A statement on whether measurements were taken from distinct samples or whether the same sample was measured repeatedly
☒	☐	The statistical test(s) used AND whether they are one- or two-sided<br>*Only common tests should be described solely by name; describe more complex techniques in the Methods section.*
☒	☐	A description of all covariates tested
☒	☐	A description of any assumptions or corrections, such as tests of normality and adjustment for multiple comparisons
☒	☐	A full description of the statistical parameters including central tendency (e.g. means) or other basic estimates (e.g. regression coefficient) AND variation (e.g. standard deviation) or associated estimates of uncertainty (e.g. confidence intervals)
☒	☐	For null hypothesis testing, the test statistic (e.g. F, t, r) with confidence intervals, effect sizes, degrees of freedom and P value noted<br>*Give P values as exact values whenever suitable.*
☒	☐	For Bayesian analysis, information on the choice of priors and Markov chain Monte Carlo settings
☒	☐	For hierarchical and complex designs, identification of the appropriate level for tests and full reporting of outcomes
☒	☐	Estimates of effect sizes (e.g. Cohen's d, Pearson's r), indicating how they were calculated

Our web collection on statistics for biologists contains articles on many of the points above.

Software and code

Policy information about availability of computer code

Data collection	Crystal diffraction data was collected at Diamond Light Source beamlines, using GDA. UV-Vis spectroscopy data was collected using Agilent Cary Win UV software.
Data analysis	COOT 0.9.8.8, CCP4 8.0.013, Phaser MR 2.8.3, Pymol 2.5, EasySpin toolbox 5.2.28, Origin Pro 9.1,GraphPad Prism 9.0, PHENIX 1.20.1-4487, PDBredo, PDBeFOLD, PDB2PQR 2.1.0, ABPS server, ChimeraX 1.3, Thermo Xcalibur

For manuscripts utilizing custom algorithms or software that are central to the research but not yet described in published literature, software must be made available to editors and reviewers. We strongly encourage code deposition in a community repository (e.g. GitHub). See the Nature Portfolio guidelines for submitting code & software for further information.

Data

Policy information about availability of data

All manuscripts must include a data availability statement. This statement should provide the following information, where applicable:

- Accession codes, unique identifiers, or web links for publicly available datasets
- A description of any restrictions on data availability
- For clinical datasets or third party data, please ensure that the statement adheres to our policy

PDB coordinates for the structures reported in this study have been deposited in the Protein Data Bank with the accession codes 5C8A [http://doi.org/10.2210/

pdb5C8A/pdb], 5C8D [http://doi.org/10.2210/pdb5C8D/pdb], 5C8E[http://doi.org/10.2210/pdb5C8E/pdb], 5C8F[http://doi.org/10.2210/pdb5C8F/pdb], 8C31, 8C32, 8C33, 8C34, 8C35, 8C36, 8C37, 8C73 and 8C76. All other source data are provided as supplementary data files.

Research involving human participants, their data, or biological material

Policy information about studies with human participants or human data. See also policy information about sex, gender (identity/presentation), and sexual orientation and race, ethnicity and racism.

Reporting on sex and gender	N/A
Reporting on race, ethnicity, or other socially relevant groupings	N/A
Population characteristics	N/A
Recruitment	N/A
Ethics oversight	N/A

Note that full information on the approval of the study protocol must also be provided in the manuscript.

Field-specific reporting

Please select the one below that is the best fit for your research. If you are not sure, read the appropriate sections before making your selection.

☒ Life sciences ☐ Behavioural & social sciences ☐ Ecological, evolutionary & environmental sciences

For a reference copy of the document with all sections, see nature.com/documents/nr-reporting-summary-flat.pdf

Life sciences study design

All studies must disclose on these points even when the disclosure is negative.

Sample size	Sample sizes were decided based on common practice in the field [e.g. crystal data collection to obtain >95% completeness]
Data exclusions	No data was excluded
Replication	Data reported from technical replicates. Independent replication attempts successfully showed an identical trend.
Randomization	Not applicable as this is not a clinical study and experiments did not need allocation to groups.
Blinding	Not applicable as this is not a clinical study.

Reporting for specific materials, systems and methods

We require information from authors about some types of materials, experimental systems and methods used in many studies. Here, indicate whether each material, system or method listed is relevant to your study. If you are not sure if a list item applies to your research, read the appropriate section before selecting a response.

Materials & experimental systems

n/a	Involved in the study	
☒	☐	Antibodies
☒	☐	Eukaryotic cell lines
☒	☐	Palaeontology and archaeology
☒	☐	Animals and other organisms
☒	☐	Clinical data
☒	☐	Dual use research of concern
☒	☐	Plants

Methods

n/a	Involved in the study	
☒	☐	ChIP-seq
☒	☐	Flow cytometry
☒	☐	MRI-based neuroimaging
